# Treatment of Club-foot by Excision of Bone from the Dorsum Pedis

**Published:** 1880-05

**Authors:** Herman Mynter

**Affiliations:** Gottingen


### TRANSLATIONS.

---

### TREATMENT OF CLUB-FOOT BY EXCISION OF BONE FROM THE DORSUM PEDIS, BY PROFESSOR KOENIG, IN GOTTINGEN.

FROM THE GERMAN BY HERMAN MYNTER, M. D.

THE author recommends a new operation for pes varo-equinus in cases in which it is impossible to cure the deformity by aid of tenotomy, and forced reduction. He has performed the operation in three cases, of which two recovered, while one died of heart disease before the wound had closed. The ages of the patients were 12, 13 and 19 years. The operation was performed strictly antiseptically, and consists in exsecting a wedge-shaped part of the metatarsal bones through longitudinal incisions on the outer part of the dorsum pedis. His conclusions are as follows:

1. A wedge-shaped excision of the dorsum pedis is the surest and least dangerous method of correcting neglected and useless club-feet, no matter whether congenital or paralytic. To amputate such feet is scarcely any longer justifiable. It is safer than violent reduction, which has been followed with gangrene.

2. The wedge-shaped excision must be made from dorsum pedis with due regard both to the adduction of the foot (pes varus) and to the hyper-extension of the foot (pes equinus) so that the base of the wedge must be relatively on the outer and dorsal, and on the dorsal side of the foot. In cases of congenital club-foot we must generally remove the head and neck of the talus and the cuboid bone, and if the foot is bent together (plantarflexion) the os naviculare. In paralytic club-foot the wedge must be exsected farther forward. Remove first a little wedge from the outer side, either with knife or chisel, and afterwards remove the bones in the middle of the dorsum pedis. It is not necessary to consider the joints except the tibio-tarsal joint.

3. Longitudinal incisions are preferable when made over the most prominent point. Transverse incisions give perhaps more space, but endanger the tendons, and favor, in paralytic feet, sloughing of the skin. If one incision is insufficient, another may be made parallel with it. It is sometimes of advantage to make tenotomy of the Achilles, and sometimes the plantar aponeurosis must be cut.

4. It is best to remove enough that the deformity may be overcome without the aid of bandages. The antiseptic treatment must be used in all its rigor; the operation is easier, if Esmarch's bandage is used.—*Centralblatt fuer Chirurgie.*

